# Predicting attention deficits and functional recovery after glioma resection through functional executive networks: insights from dynamic properties

**DOI:** 10.1007/s11060-025-05079-w

**Published:** 2025-06-10

**Authors:** Francesca Saviola, Luca Zigiotto, Jorge Jovicich, Silvio Sarubbo

**Affiliations:** 1 Center for Mind/Brain Sciences, University of Trento, Rovereto, Trento, Italy; 2 Neuro-X Institute, Ecole Polytechnique Fédérale de Lausanne (EPFL), Lausanne, Switzerland; 3 Department of Neurosurgery “S. Chiara” University Hospital, Azienda Provinciale per i Servizi Sanitari (APSS), Trento, Italy; 4 Center for Medical Sciences (CISMed), Department of Cellular, Integrative and Computational Biologi (CIBio), University of Trento, Trento, Italy

**Keywords:** Gliomas, Dynamic functional connectivity, Functional recovery, Postsurgical attentional deficit, fMRI

## Abstract

**Background:**

Postoperative short-term attentional and executive dysfunctions are common after brain tumor resection, significantly impacting patients’ quality of life and functional recovery. This longitudinal cross-sectional study investigated whether presurgical functional dynamics of key brain networks supporting executive functioning could predict postoperative neuropsychological outcomes, providing insights into temporary deficits and recovery trajectories.

**Methods:**

Twenty-two patients with gliomas underwent resting-state fMRI scans before and three-months after surgery, along with neuropsychological assessments conducted before, one week and three months after surgery. Co-activation pattern analysis (CAPs) characterized functional dynamic properties of executive networks, including the Fronto-parietal (FPN). Temporal network properties - stability, integration, and centrality- were examined longitudinally. Descriptive and predictive multivariate analysis explored associations between network dynamics and cognitive functioning.

**Results:**

Immediate post-surgical attentional deficits were associated with pre-surgical FPN properties, revealing dynamic activation patterns predictive of short-term deficits. These temporal properties not only predicted the severity and persistence of early deficits, but also offered valuable insights in the longitudinal progression of attentional performance otherwise neglected. Importantly, by three months post-surgery, neuropsychological profiles and network dynamics returned to pre-surgical baseline, highlighting the transient nature of the deficits beyond treatment strategies.

**Conclusions:**

Our study demonstrates that presurgical dynamic properties of intrinsic executive networks alone can predict short-term postoperative neuropsychological outcomes. This predictive ability offers critical value for patients, families and clinical teams by emphasizing the temporary nature of the deficits and enabling early, personalized interventions. These findings emphasize the potential for using intrinsic brain activity dynamics as a tool for guiding postoperative recovery planning and alleviating concerns about temporary postoperative cognitive impairments.

**Supplementary Information:**

The online version contains supplementary material available at 10.1007/s11060-025-05079-w.

## Introduction

Postoperative attentional and executive dysfunctions are common following brain tumor resection, affecting both patients’ quality of life and functional recovery [1, 2]. Indeed, after surgery the immediate outcome in the attentive domain in glioma patients can sometimes reveal cognitive deficits, even in the absence of clear presurgical insufficient performance [3]. Importantly, also during awake brain surgery the correct monitoring of several manifestations of attention and executive functions is challenging [4]. Therefore, the ability to predict the occurrence of this type of post-surgical deficits could be crucial for improving clinical practice, not only for surgical treatments, but also for further treatments more focused on illness progression such as radiotherapy [5, 6]. Despite advancements in the field, accurately predicting temporary or permanent post-surgical deficits or recovery in attention and executive function at the individual level remains demanding with current technology. Consequently, whether advanced functional neuroimaging techniques, in the absence of any other reliable clinical marker, can help anticipate such deficits prior to surgical resection remains an open question.

Large-scale functional brain networks are known to be involved in executive function processes, such as attention, working memory, inhibitory control, cognitive flexibility and shifting. Previous studies, both in healthy [7, 8] and glioma patients [9–13] demonstrated how functional executive connections can sustain cognitive performance in the attentive domain. Specifically, two main neural networks seemed to be involved in several executive and attentive performances: the fronto-parietal network (FPN), a flexible network strictly related to goal-directed behavior or or cognitive control [14] and the dorsal attention network (DAN), engaged during integrative externally directed attentional tasks [15]. Attentional functional networking can be reconstructed by looking at statistical dependencies in activity between distant but functionally interrelated regions in the brain, the so-called intrinsic functional connectivity [16]. Furthermore, given the inherent brain properties of dynamism, recent frameworks exhibited how spatiotemporal fluctuations of blood oxygenation level dependent (BOLD) signal strongly characterize time-varying properties of cognition at rest [17], posing it as an extremely relevant methodology in the context of non-stationary cognitive shifting abilities such as attention. Consequently, longitudinal dynamic plastic changes of these intrinsic functional networks could be informative about the resilient support of cognitive performance, especially the attentive one. In this work, we took advantage of dynamic intrinsic functional connectivity estimations, previously demonstrated to be clinically relevant [18]. We aimed at characterizing the pathological functional temporal profile of attentive networking and testing their potential as a clinical tool to predict these cognitive performance’s changes.

### Aims

Here, we combine neuroimaging findings from intrinsic functional magnetic resonance imaging (fMRI) with neuropsychological assessment, pre- and post-surgery, to predict–before the actual surgery – the cognitive outcome. This approach may facilitate the development of more precise and tailored therapeutic interventions and thus ultimately enhance a patient’s functional recovery and attentional performance gains. We first mapped longitudinal dynamics of attention and executive functional networking, such as the FPN and the DAN, across subjects. Then we associated patterns of temporal properties with the neuropsychological longitudinal performance highlighting how impaired patients (i.e. attentive/executive deficits at presurgical presence or at immediate post-surgical onset) are characterized by specific brain dynamic patterns. Lastly, we hypothesized that enabling the prediction of post-surgical attentive outcome based on the pre-surgical clinical and executive network dynamic features would allow not only to identify high-risk patients (i.e. potentially exhibiting post-surgical attentional deficits) affected by low- or high-grade gliomas (LGGs and HGG, respectively), but also to preventively warn the neurosurgeon for a more careful preservation of executive networks’ areas thus improving clinical long-term prognosis.

## Materials and methods

### Participants

A total of 22 gliomas patients were recruited at Santa Chiara Hospital in Trento, Italy. Approval for this study was obtained by the Ethical Committee of the Azienda Provinciale per i Servizi Sanitari (APSS, Neusurplan project, authorization ID A734). Subjects (16 males, age 47.8 ± 14.8 years, 11 right-hemisphere tumors) had a diagnosis of gliomas of different malignancy grades, with 13 high-grade (HGG) and 9 low-grade gliomas (LGG) patients [19]. See Table 1 for more demographic and clinical information. Resting-state fMRI (rs-fMRI) and T1-weighted longitudinal images were acquired on a 1.5T MRI system along with a comprehensive neuropsychological assessment with a specific focus on attentive and executive functions [20]. Participants kept eyes open during scanning. See Supplementary Materials for details [21].

**Table 1:** Demographic and clinical characteristics of the sample. Further details about surgery and clinical care-related outcomes are reported in the supplementary materials

Patients	Brain tumor types			Statistic	*p*-value
	Low grade gliomas	High grade gliomas	Total		
Age (mean ± SD)	46 ± 15	47 ± 15	48 ± 15	t-value:-1.87	0.07
Gender (# males)	7	9	16	*χ*2: 0.43	0.67
Tumor lateralization (# Left)	6	5	11	*χ*2: -1.29	0.21
Tumor WHO grade (# patients)	[grade-1: 1; grade-2: 8]	[grade-3: 4; grade-4: 9]	N.A.	N.A.	N.A.
Tumor volume cm^3^ (mean ± SD)	28.2 ± 29.9	32.6 ± 30.2	28.1 ± 28.9	t-value=-1.74	0.09
Awake surgery (# patients)	9	8	17	N.A.	N.A.
IDH-1 mutation (# patients)	0	2	2	N.A.	N.A.
MGMT methylation (# patients)	0	6	6	N.A.	N.A.
Radiotherapy [(# patients (week mean, SD)]	0	9	13	N.A.	N.A.
Proton therapy [(# patients (week mean, SD)]	0	4	4	N.A.	N.A.
Chemotherapy [(# patients (week mean, SD)]	0	12	12	N.A.	N.A.

### Dynamic network analysis

We applied a framewise dynamic functional connectivity method (co-activation patterns, CAPs [22]; https://github.com/MIPLabCH/iCAPs., see Supplementary Materials for details) to assess longitudinal functional plasticity changes in intrinsic executive networking. While other functional large-scale networks are also implicated in cognitive control and attentional switching, the FPN and DAN were selected as primary networks of interest due to their direct and well-established involvement in externally directed attention, goal-oriented behavior, and executive functioning [14, 15]. While other functional networks interact dynamically with the FPN and DAN, the latter are most consistently engaged during attention-demanding tasks. Focusing on these networks allowed us to specifically target the neural substrates most relevant to our clinical and cognitive outcomes, while acknowledging that future studies could extend this approach to include whole-brain networking.

Using seeds from the FPN and DAN, we extracted co-activations and co-deactivations patterns in BOLD signals [23]. Consensus clustering was performed by repeatedly applying k-means clustering to resampled subsets of the data and evaluating the consistency of clustering assignments, ensuring the robustness of the identified CAPs. This revealed k = 4 as the best-fitting model order for both seed sets. For each CAP, temporal metrics were computed across sessions: IN-degree, OUT-degree, resilience, betweenness centrality, and occurrences [24, 25]. Longitudinal changes (Δ) in these properties were calculated as the difference between post- (3-month) and pre-surgery values.

### Neuropsychological assessments

We analyzed longitudinal neuropsychological assessments, focusing on executive function domains at different timepoints (presurgical, one-week post-surgical, and three-months follow-up). Other cognitive domains are reported elsewhere [4, 21, 26]. We included scores of attentional and executive functions, tested using attentional matrices and Trial Making Test (TMT: A, B, B-A scores) [27]. Percentage of cognitive deficits for each test is reported in Supplementary Table S1. A dichotomous classification was calculated by evaluating each patient’s neuropsychological scores one week after surgery; if any score fell below the established clinical cutoff for impairment, the patient was classified as having a cognitive deficit, whereas patients with all scores within the normal range were classified as not having a deficit. Neuropsychological assessment was conducted using a validated battery of tests for LGG and HGG [3, 4, 26]. Testing was performed at specific timepoints relative to surgery and MRI scanning. Scores were adjusted for age and education (following [28]);, and pathological scores were determined using a dichotomous classification. We also calculated Δ scores in attentional and executive functions between pre-surgery and 3-months follow-up evaluations. Detailed information on assessment timing, score adjustments, and deficit classification can be found in the Supplementary Materials.

### Statistical analysis

Statistical analyses were performed using Matlab 2019b and R (v3.6.0). Neuropsychological scores and temporal properties of dynamic networks [29] were compared between sessions using paired t-tests. Pearson’s correlation coefficient assessed relationships between changes in executive function scores and CAP temporal occurrences. Partial least squares correlations (PLSC, behavioral grouped version [30, 31]) explored multivariate connections between executive FPN or DAN CAPs temporal measures and executive domain deficit scores. In this context, each latent component (LC) represents a pattern of association that links specific changes in the temporal properties of FPN CAPs or DAN CAPs with changes in attentional and executive test scores across patients. A significant LC indicates that a particular combination of network features covaries with a particular combination of behavioral outcomes, thus capturing the main axes along which brain network dynamics and executive/attentional cognitive recovery are related in our cohort. Linear and mixed models were used to predict postoperative attentive performance based on dynamic properties of executive networking. In simple terms, these linear models were used to see if patterns in brain network activity could help predict how well patients would perform on attention and executive function tests, both before and after surgery. Detailed methods are available in the Supplementary Materials.

## Results

### Longitudinal executive and attentive profiles

Supplementary Table 1 shows the group average executive and attentive scores at the various treatment stages. The two-sample paired t-tests revealed that immediately post-surgery (i.e. 1 week after surgery) patients significantly decreased (*p* < 0.05; t-value_TMTA_: -2.4, t-value_TMTB_: -3.6, t-value_TMTB−A_: -3.1) executive and attentive performance scores (i.e. TMT), regardless of tumor grade and hemisphere, whereas attentional matrices showed no significant changes. The three-month follow-up time point showed no significant differences in executive and attentive performance (i.e. attentional matrices and TMT, *p* > 0.05) relative to the presurgical stage, regardless of tumor grade and features.

### Longitudinal temporal dynamics of executive networks

**Fig. 1:**
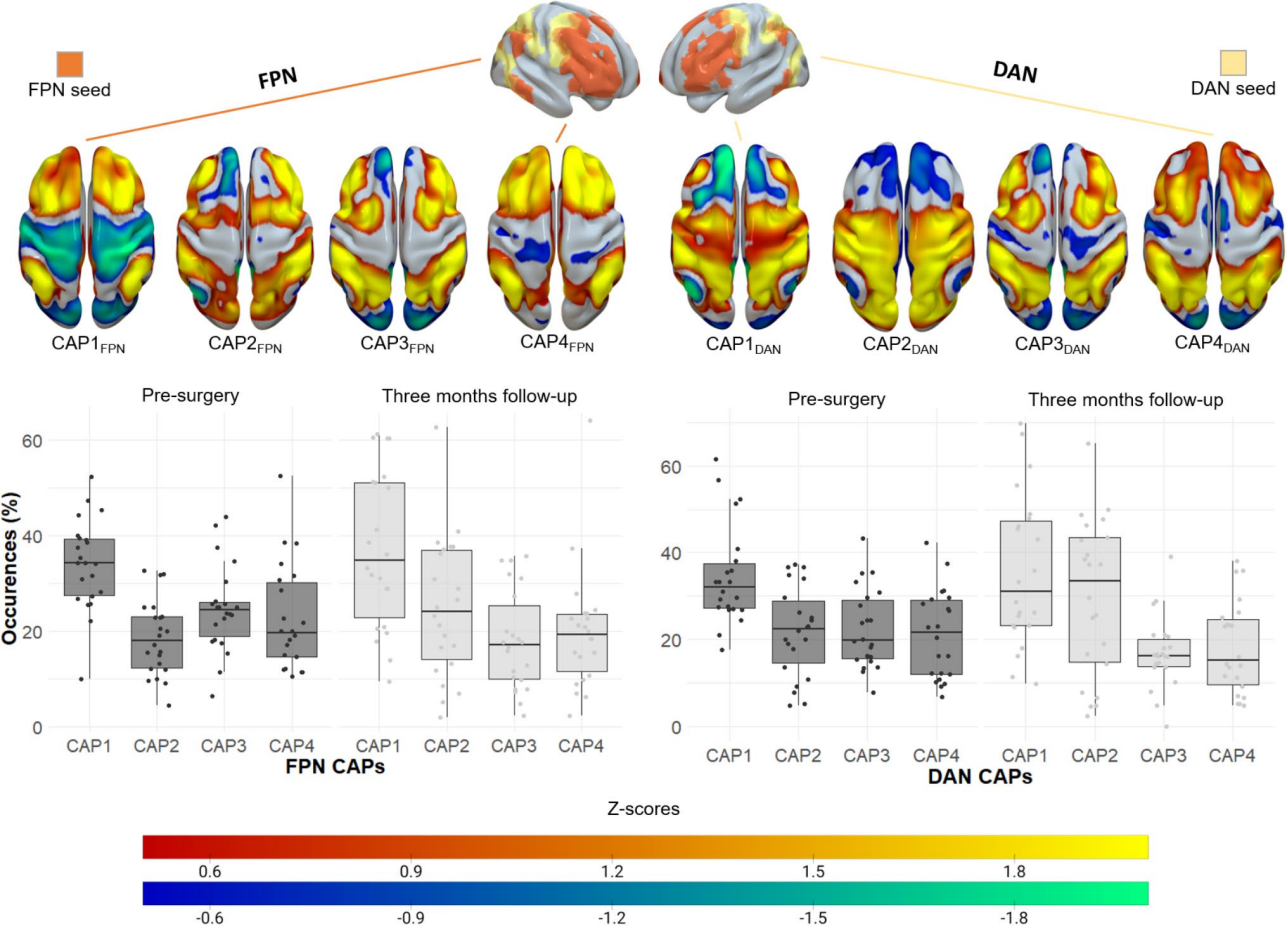
Longitudinal fronto-parietal and dorsal attention networks dynamism in gliomas. First row, anatomical placement of the fronto-parietal (FPN, left) and dorsal attention (DAN right) seeds from the seven networks parcellation (Yeo et al., 2011). Second row, four Co-activation patterns (CAPs) spatial distribution characteristics of all subjects for the two timepoints extracted from the corresponding seeds. Third row, boxplot of CAPs dynamic features over the time course (i.e. percentage of the time each subject is spending in each CAP over the time course)

Figure 1 illustrates the CAPs for k = 4 using FPN and DAN seeds, which represent various states of the executive network. For the FPN CAPs, CAP1_FPN_ shows significant activation in frontal and bilateral parietal regions, with deactivation in somatosensory and visual areas. CAP2_FPN_ primarily activates dorso-lateral frontal regions along with greater involvement of the temporo-parietal areas. CAP3_FPN_ reflects activation similar to that of the DAN, with deactivation occurring in occipital regions. Finally, CAP4_FPN_ exhibits strong activation within fronto-parietal regions, often referred to as the “*central-executive*”. In terms of the DAN CAPs, CAP1_DAN_ is characterized by substantial somato-sensorial activation in the temporal and parietal lobes, accompanied by deactivation in bilateral medial cingulate regions. CAP2_DAN_ shows strong activation in temporo-parietal regions, including the dorsal portion of the occipital lobe. CAP3_DAN_ resembles the DAN with activation in the intraparietal sulcus and frontal eye-fields, while CAP4_DAN_ activates similar regions as CAP3_DAN_ but to a lesser extent. Overall, there were no statistically significant differences in longitudinal properties across subjects for either the FPN or DAN states (*p* > 0.05).

### Temporal properties of fronto-parietal network are associated with attentional and executive cognitive profile

**Fig. 2:**
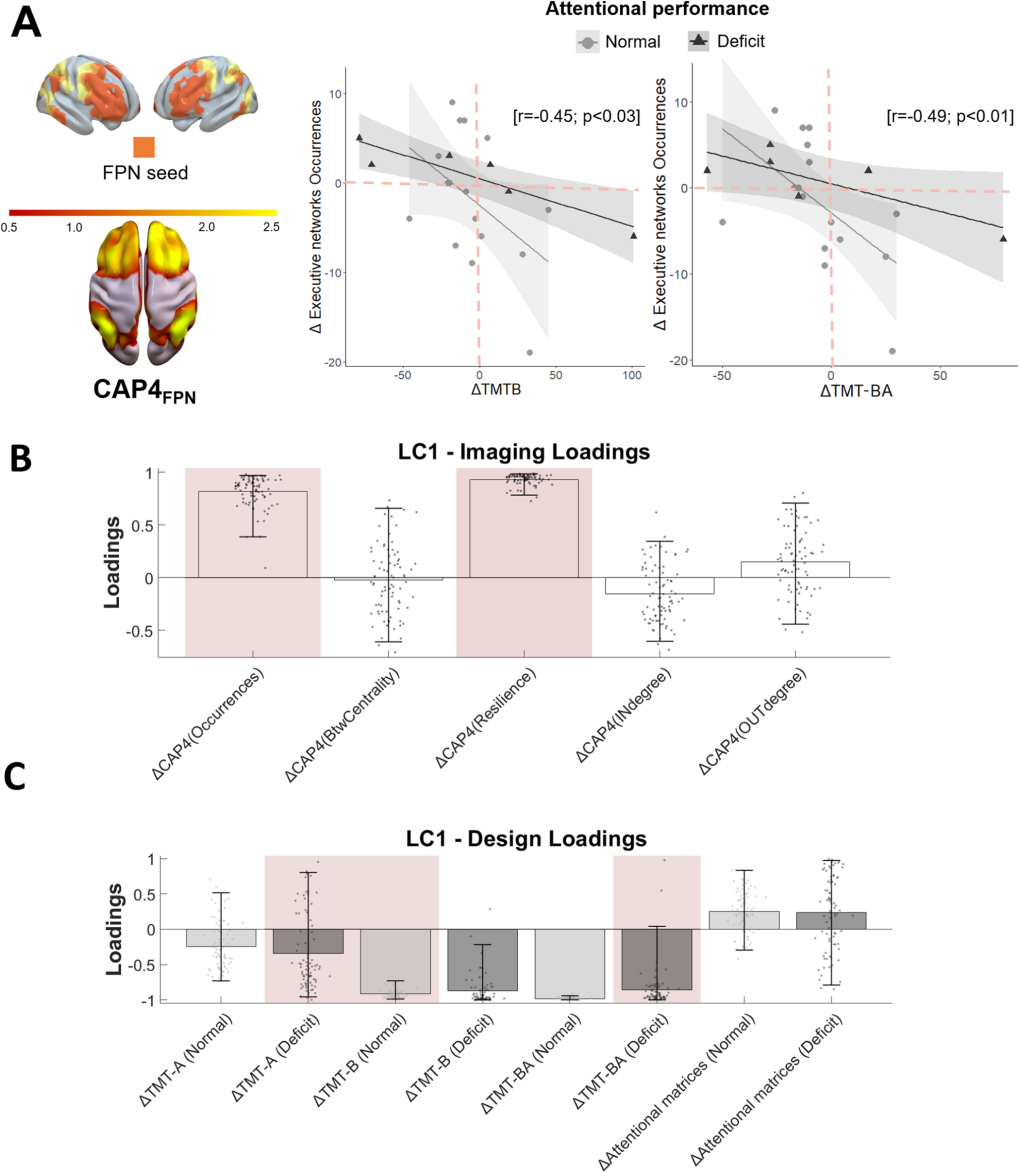
Functional signature of attentional cognitive outcome through fronto-parietal dynamism. (**A**) Correlation between changes in attentional performance and changes in fronto-parietal CAP duration between the 3-months follow-up and the presurgical stages (ΔTMTB and ΔTMT-BA calculated by computing the difference between the performance scores between the evaluation after (3 months follow up) and before surgery). (**B**) and (**C**) Significant Latent Component (LC) identified with grouped behavioral Partial Least Squares Analysis explaining differing effects of covariance in patients with different attentive performance disregarding absolute group differences. Errorbars indicate bootstrapping 5th to 95th percentiles, robust results from permutation testing are highlighted by pink boxes

Neuropsychological assessment of executive functions showed no significant differences across attentional deficit groups pre-surgically and at 3 months follow-up (*p* > 0.05). Analysis of associations between changes in CAPs temporal properties and attentional performance revealed that only CAP4_FPN_ was significantly correlated with cognitive profile (Fig. 2A). Longitudinal variations in TMT-B and TMT-BA scores were negatively associated with changes in CAP4_FPN_ temporal duration. This indicates that for patients with pre-surgical attention deficits, decreased FPN temporal duration (negative Δ) was associated with impaired post-surgical cognitive performance, while increased or unchanged FPN occurrence (positive or near-zero Δ) was associated with cognitive recovery at three months post-surgery.

To better understand the association between CAP4_FPN_ temporal properties and overall attentive profile, multivariate analysis was employed (behavioral grouped PLSC). The analysis revealed one significant LC (*p* = 0.006; Supplementary Digital Content) with a strong correlation between behavioral and imaging scores (*r* = 0.80). Figure 2B displays CAP4_FPN_ temporal features weights, while Fig. 2C shows behavioral weights. Results indicate that increased occurrences and resilience of CAP4_FPN_ state post-surgery are associated with lower Δ TMT-A values in the attentive impaired group, and lower Δ TMT-B and higher Δ attentional matrices values in the normal executive functioning group.

### Longitudinal attention profile changes: the role of fronto-parietal network dynamics

**Fig. 3:**
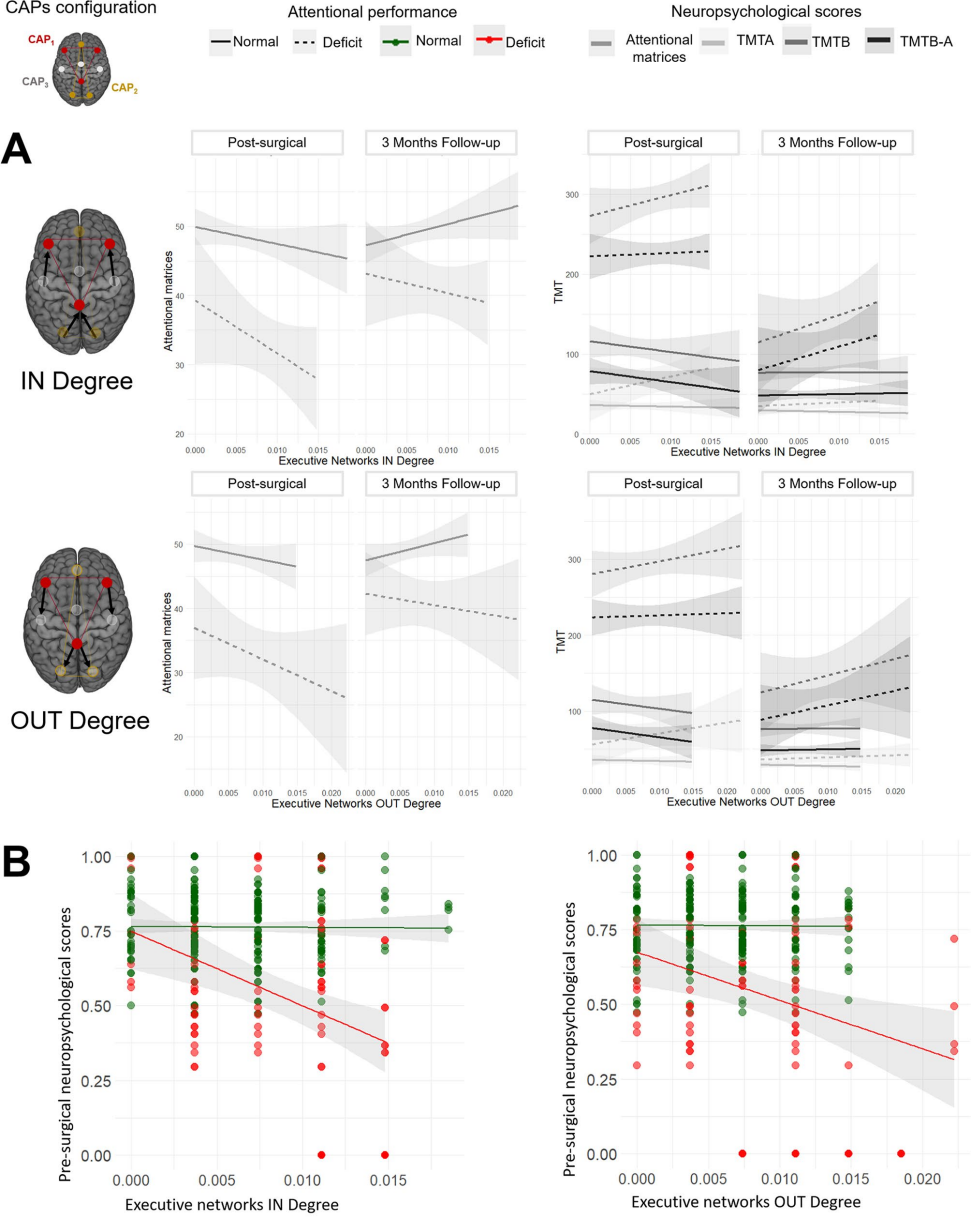
Dynamic networking as indicator of post-surgical attentional deficits. (**A**) Linear fitting of immediate post-surgical or three-months follow-up attentional and executive performance, displayed as a function of pre-surgical IN Degree properties of FPN and pre-surgical OUT Degree properties of FPN for each grouped neuropsychological test. (**B**) The scatterplots depict the association between pre-surgical dynamic rs-fMRI properties of the FPN (x-axes) and pre-surgical performance in attentional and executive tasks (y-axis). FPN dynamics are represented by IN Degree properties (probability of transitions from other dynamic states to the FPN, left) and OUT Degree properties (probability of transitions from the FPN towards other dynamic states, right). Neuropsychological test scores are presented in normalized z-score forms. The colored dots distinguish two groups of patients: those who, 1-week post-surgery, showed deficits in at least one neuropsychological test (red dots) and those without deficits (green dots). Separate linear fits of pre-surgical data in these two groups are shown

A linear mixed model examined associations between attentive performance and executive network dynamics (Supplementary Materials: Model 1). TMT scores showed significant fixed effects (*p* < 0.05) for post-surgical deficit and FPN states’ betweenness centrality. Significant interactions (*p* < 0.05) were found between attentional deficit, FPN state properties, and time. FPN states’ stability predicted attention deficit, while higher IN- and OUT-degree (i.e. how many transitions the states carried out) values explained deficit presence. Model 2 (Supplementary Materials) tested if presurgical dynamics predicted attentional deficits, showing significant effects (*p* < 0.05) for transition properties. Model 3 (Supplementary Materials) revealed CAP stability properties’ relevance for prediction. Models 4 and 5 (Supplementary Materials) predicted post-surgical outcomes. Model 4 showed significant effect (*p* < 0.05) for IN-degree. Model 5 revealed significant effects for FPN transitions in executive scores. Figure 3 illustrates pre-surgical behavioral and functional data differentiating patients with and without post-surgery attention deficits. Scatterplots of normalized pre-surgical attentive and executive scores in relation to functional dynamic metrics (in-degree and out-degree) revealed distinct distributions between patients with post-surgery attention deficits (red dots) and those without (green dots). Notably, reduced short-term post-surgery attentive cognitive scores were strongly associated with pre-surgical increases in both in-degree and out-degree indices of the FPN.

## Discussion

In this study, we investigated dynamic features of intrinsic executive and attentive network brain activity in glioma patients, focusing on the identification of presurgical functional signatures with a predictive value of post-surgical attentive deficits. These markers are crucial for patient recovery and for guiding both surgical and non-surgical oncological treatments.

To the best of our knowledge, this is the first study investigating the association between the dynamics of large-scale executive and attentional brain networks and post-surgery attentional deficits in glioma patients. We took advantage of the CAPs framework [25] to estimate dynamic brain functional properties and move beyond static functional connectivity analysis of resting-state activity [21]. Our findings highlighted alterations in brain network dynamics related to attentive deficits, especially affecting the stability of the executive network, and identified these alterations as predictors of post-surgical cognitive performance recovery.

### Understanding glioma pathophysiology: effects on attention and executive brain network dynamics

In our study, we investigated the longitudinal cognitive outcomes of patients with and without post-surgical attentional deficits, who importantly showed a complete recovery three months after surgery. In this context, our aim was to understand and identify specific early changes in the functional dynamics of networks associated with attentional performance, such as the FPN and the DAN, as no other clinical or demographic variables showed a significant effect on post-surgical cognitive outcomes. Importantly, single-subject network dynamics did not significantly change between the presurgical state and the three-month follow-up. This suggests that the overall longitudinal dynamic properties of the FPN and DAN are not inherently affected by surgical resection, as previously reported by static connectivity analyses [21]. Nevertheless, the presence of immediate post-surgical attentional deficits in our cohort was found to be negatively associated with changes in both occurrence and resilience properties of a specific FPN state relative to the presurgical stage. Previous studies have demonstrated that preserving the integrity of functional brain networks after brain surgery [21] is crucial for the recovery of several cognitive abilities, such as memory. However, several studies have reported a reduction in FPN occurrences both statically and in other types of dynamic connectivity analyses in gliomas [13, 32–38]. Furthermore, research on dynamic connectivity and cognitive performance in glioma patients has indicated that the absence of stable reconfiguration properties within the FPN network serves as a negative prognostic indicator for functional recovery [39, 40]. Notably, while confirmatory analyses were also performed for the DAN, our results showed that the dynamic properties of the FPN were substantially more predictive of post-surgical cognitive outcomes. This distinction may reflect the central and flexible role of the FPN in executive control, integrating information across multiple cognitive domains and supporting goal-directed behavior, whereas the DAN is more specifically engaged in externally oriented attentional processes. Thus, the domain-general, adaptive functions of the FPN may render its dynamics particularly relevant for predicting broader aspects of cognitive recovery following glioma resection.

Taken together, these findings suggest that plastic reorganization of FPN temporal properties—specifically longitudinal changes in network occurrences—may be closely linked to changes in patient cognitive performance. This underscores two critical notions: first, aberrant dynamic reconfiguration of the FPN, particularly its temporal stability [41], may lead to impaired cognitive compensation three months post-glioma resection; second, these results highlight the need to spare critical hubs of this network during surgery to facilitate cognitive improvements observed in our cohort, which showed no pathological scores at least three months after surgery.

Considering the FPN as a key hub for cognitive flexibility, task-shifting, and goal-oriented behavior, our findings support the idea that modifications in its temporal properties could significantly impact cognitive executive functioning and plasticity in gliomas.

### Functional dynamics of pre-surgical intrinsic executive networks predicts post-surgical attentive deficits

Recent evidence challenges the notion of a reliable anatomo-functional pre-surgical predictor for executive dysfunction [42]. Therefore, in aiming for a better understanding of the topography of executive function performance, we examined the longitudinal plastic reorganization of temporal network properties within the FPN to characterize presurgical patterns contributing to short-term post-surgical attentional deficits. Our study demonstrated that presurgical intrinsic network dynamic properties of the FPN were predictive of post-surgery attentional performance, both immediately after surgery and at the three-month follow-up.

In this context, we emphasize the significance of two key aspects of FPN dynamics: stability (as indicated by occurrences) and temporal flexibility (represented by IN- and OUT-degree) in determining pre-existing attentional deficits and their persistence post-surgery. Specifically, a less stable and constantly switching FPN network is associated with attentional deficits that are present before surgery and continue to persist afterward. Importantly, our results are unrelated to tumor grade (i.e., high- or low-grade) or lateralization (i.e., left or right brain hemisphere), highlighting the role of cortical networks and subcortical connections over lesion or tumor location [43].

Our findings offer novel and clinically relevant insights that can inform treatment decisions for patients. Given the significant relevance of FPN networking highlighted in our study, it is crucial to incorporate it into considerations when determining therapeutic options aimed at minimizing post-surgery recovery time for patients.

Indeed, predicting and recognizing that patients with distinct pre-surgical FPN dynamic characteristics who are unlikely to experience attentional deficits or who are poised for swift recovery can offer valuable guidance for radiotherapists. This information can help in determining the optimal radiation dose to administer near the surgical cavity and tumor. Conversely, a more targeted radiation treatment that focuses specifically on the surgical cavity while minimizing exposure to neighbouring regions should be carefully considered for patients at risk of retaining post-surgery deficits. Additionally, our study provides valuable predictive information about a patient’s cognitive status following surgery, which can be crucial for their understanding and preparation. However, the relatively small sample size represents a limitation of this study, potentially affecting the generalizability and robustness of the findings. Further longitudinal studies are essential to validate the subject-specific predictive potential of post-treatment cognitive outcomes derived from pre-treatment intrinsic dynamic functional connectivity properties.

### Implications in the clinical practice for patients care, rehabilitation and treatment

In this context, investigating the relative longitudinal changes in attentive performance holds significant potential benefits. Our findings offer novel and clinically relevant insights that can inform treatment decisions for patients. The emphasis of our study on the crucial role of FPN connectivity, as revealed by our analyses specifically targeting executive-only networking, underscores the imperative to incorporate this network into therapeutic strategies aimed at optimizing patients’ post-surgical recovery trajectories.

Indeed, identifying patients with distinct presurgical FPN dynamics who are less likely to experience attentional deficits or are primed for rapid recovery can provide crucial guidance to radiotherapists, informing decisions on optimal radiation dosage near the surgical cavity and tumor site. Additionally, our study provides valuable predictive information about a patient’s cognitive status following surgery, which can be critical for their understanding and preparation, as well as for obtaining informed consent.

Recognizing the onset of post-surgical attention deficits, even if temporary and reversible, is essential for initiating targeted cognitive interventions early in the clinical care process. This proactive approach can significantly reduce deficits within weeks, accelerating cognitive recovery and facilitating an earlier return to work. Such timely intervention is particularly vital given the potential cognitive impact of adjuvant therapies like chemotherapy and radiotherapy [44, 45]. By promptly addressing attention deficits, we can mitigate their effects and potentially offset additional cognitive challenges posed by subsequent treatments.

Importantly, even when performance isn’t pathological, the observed decline in patients’ attentional abilities (Fig. 2A) can detrimentally affect their perception of Health-Related Quality of Life (HRQoL) [46]. Therefore, predicting attentional worsening and implementing early interventions could not only improve cognitive outcomes but also significantly enhance patients’ overall HRQoL, underscoring the comprehensive benefits of this approach.

However, while our study focuses on adult glioma patients, evidence from pediatric neurosurgery suggests children often show faster and more pronounced cognitive recovery after surgery. Studies report dynamic changes in brain network variability and cognition in children following neurosurgical procedures, highlighting greater neuroplasticity compared to adults. These developmental differences in recovery trajectories underscore the importance of considering age when interpreting post-surgical cognitive outcomes and tailoring rehabilitation strategies [47, 48].

## Conclusions

Our study reveals that post-surgical attentive performance in glioma resection patients is linked to presurgical intrinsic network dynamics, particularly within the frontoparietal network, independent of glioma grade or lateralization. Executive function deficits appear to stem from altered temporal persistence of highly co-activated frontoparietal network nodes. These insights suggest that interventions targeting the restoration of robust functional network occurrence, such as brain stimulation, could potentially prevent or ameliorate surgery-induced attentional impairments. Our findings underscore the importance of examining pre-surgical intrinsic dynamics of complex cognitive networks to enhance patient prognosis and optimize outcomes.

## Electronic supplementary material

Below is the link to the electronic supplementary material.

Supplementary Material 1

## Data Availability

Data will be made available in de-identified format upon request to the authors.

## Declarations

### Previous presentations

A preliminary part of this work was recently accepted as a short oral presentation at the International Society for Magnetic Resonance in Medicine 2024 annual meeting. The manuscript has not been published elsewhere and is available as a preprint on medRXiv (DOI: 2024.05.03.2430657 8v1).
