# Supplementary material for: Predicting attention deficits and functional recovery after glioma resection through functional executive networks: insights from dynamic properties
Source: J Neurooncol. 2025 Jun 10;174(2):459–69. doi: 10.1007/s11060-025-05079-w (PMC12209002; doi:10.1007/s11060-025-05079-w)

**Supplemental Materials**

Participants

Patients were retrospectively selected for this study based on the following criteria: 1) no previous resection of recurrent lesions; 2) availability of pre-operative rs-fMRI and structural MRI images; 3) Italian mother tongue.

MRI acquisition

Resting-state fMRI parameters: 2D T2*-weighted gradient-echo EPI, TR=2600ms, 4×4×4mm^3^ voxels, TE=45ms, FA=87°, FOV=256×256mm^2^, 33-35 slices, ASSET=2, AC-PC acquisition, 12 minutes (275 volumes). T1-weighted parameters: IRGE, TR=10.6ms, 0.5×0.5×1.0mm^3^ voxels, TE=4.23ms, TI=450ms, FA=12°, FOV=256×256mm^2^, 156-192 slices, ASSET=2. The same MRI acquisition was repeated twice, both before and after surgery (three-months follow up), to assess the changes in functional brain networking and evaluate the surgical outcomes.

MRI pre-processing

The pre-processing steps of rs-fMRI were preceded and followed by off-line quality check through visual inspection of temporal signal-to-noise ratio and standard deviation maps (for a detailed explanation of the steps see previous work^1^). Rs-fMRI and structural T1-weighted images were pre-processed with the Statistical Parametric Mapping SPM12 software (https://www.fil.ion.ucl.ac.uk/spm/software/spm12/). After DICOM to Nifti conversion of all scans, the first 10 seconds of the rs-fMRI acquisition were removed for a steady state signal. Pre-processing included the following steps: (1) slice timing and head motion correction; (2) co-registration of the T1-weighted anatomical image to the rs-fMRI time series; (3) T1-weighted image segmentation to grey matter, white matter and cerebro-spinal fluid; (4) rs-fMRI temporal filtering (median, 4th order linear detrending and 2nd order low pass filtering, Butterworth f<0.1 Hz); (5) regression from the rs-fMRI time series of 6 head motion parameters and white matter and cerebro-spinal fluid signals; (7) normalization to standard MNI template space; (8) spatial smoothing with 2 voxels Full Width Half Maximum Gaussian kernel size.

Neuropsychological assessments

Patients underwent a longitudinal neuropsychological assessment (before surgery, one-week after surgery and three months follow-up). The full list of test and cognitive domains assessed included:

- **Language:** object naming^2^, action verb naming^3^, semantic fluency^4^, phonemic fluency^4^;
- **Memory**: short-term verbal memory (verbal digit span^5^), short-term visuo-spatial memory (Corsi span^5^), long-term verbal memory (Rey Auditory Verbal Learning Task immediate and delayed recall^6^), long-term visual memory (Rey-Osterrieth complex figure/Modified Taylor complex figure delayed recall^7,8^);
- **Attention**: attentional matrices^9^, Trail Making Test part A^10^;
- **Executive functions:** Trail Making Test part B^10^, phonemic fluency^4^, Stroop test^11^;
- **Visuo-spatial abilities:** Apples cancellation test^12^, line bisection test^13^;
- **Apraxia:** constructional apraxia (Rey-Osterrieth complex figure/Modified Taylor complex figure immediate reproduction^7,8^;
- **Non-verbal intelligence:** Raven’s Coloured Progressive Matrices14;

Here we focused on results in the executive function domains for different timepoints (i.e. presurgical, one-week post-surgical and three-months follow-up), whereas others are reported elsewhere^1,15,16^. For the purpose of this study and given the entire sample availability, we included in the analyses scores of attentional and executive functions, tested using attentional matrices and Trail Making Test (A, B, B-A scores) The percentage of cognitive deficit, i.e., pathological scores, are reported for every test in Table S1. Neuropsychological assessment was conducted with a validated battery of tests widely used for LGG and HGG as previously described and performed before surgery (12.96±9.34 days), 1 week after surgery (before hospital discharge), subsequently after 3 months before or after MRI scanning to be associated with neuroimaging measures. Neuropsychological scores were adjusted for age and education^17^, as a standard clinical practice in Italy (see for instance^18^). This allows to compare scores between subject of different ages and education level, reducing at the same time variables that need to be considered in the analyses; pathological scores, to be later associated with dynamism of functional networks, were then calculated using a dichotomous classification based on the presence or absence of a cognitive deficit at the evaluation before hospital discharge (i.e. 1 week after surgery) in at least one of the neuropsychological scores, i.e. a score under cut-off (pathological) or in the normal range of performance. Furthermore, Δ scores in attentional and executive functions were calculated by computing the difference between the performance scores at each test between the evaluation after (3 months follow up) and before surgery, to be then later associated with neuroimaging findings.

Surgical procedure

Volumetric T1 with gadolinium and T2/Flair (for LGG) images, merged with the tractography and functional reconstructions of the critical cortico-subcortical structures, was used for neuro-navigation for each patient both in awake surgery and in general anaesthesia. For awake surgery, the cortical and subcortical mapping was performed at 60Hz, 1ms duration and 2-4mA of amplitude, as previously reported^19,20^. The threshold was set eliciting speech arrest at the level of the ventral premotor cortex (VPMC), regardless of tumor laterality. During awake surgery a customized intraoperative neuropsychological monitoring was performed for each patient. Surgical resection was therefore stopped when functional responses were elicited from the cortical and subcortical stimulation of eloquent structures. Depending on the lesion lateralization and location (see Figure S4 for probability maps of tumor locations), the following tasks were performed during awake surgery: counting (0-10) and motor task, object naming, verb generation, reading and comprehension, palm-pyramid-tree test (PPTT), Stroop test, line bisection, the modified version of “reading the mind in the eyes'', as previously reported^20-22^.

Dynamic functional connectivity analysis

CAPs analysis was performed by concatenating pre- and post-surgical timepoints (i.e. increasing the dataset’s size and diversity, leading to more stable and generalizable patterns^23^) of every subject, and then by extracting co-activations and co-deactivations patterns in the BOLD signal respect to the seeds, for limited periods of the time course, with temporal clustering aiming at detecting fluctuations of the executive networks with their components (fronto-parietal network (FPN) and dorsal attention network (DAN)). For each set of seeds, we extracted and z-transformed the seed BOLD time course and selected the top 10% time points with the highest activation. Consensus clustering revealed k=4 as the best-fitting model order for both seed sets. By using k-means clustering^24^, using the cosine distance and random initialization of the algorithm, the spatial z-maps of the four CAPs for the FPN and the four CAPs for DAN were obtained. For each detected CAP, temporal metrics were computed across temporal sessions by computing the back-project of the group networking at the single-subject level: (i) IN-degree: how likely a CAP is visited by any other one, (ii) OUT-degree: how likely a CAP exits towards any other one, (iii) resilience: the likelihood to remain in the same configuration towards the time course, (iv) betweenness centrality: how important a CAP is regarding the shortest paths between other CAP pairs, (v) occurrences: how much a CAP re-occurs over the time course^25,26^.

Partial least squares correlation (PLS-C)

The PLS-C methodology is a multivariate statistical method that finds the latent variables, or mutually orthogonal, weighted linear combinations of the original variables in the two datasets that have the highest degree of correlation with one another. In the current analysis, one dataset represent the temporal properties of relevant FPN-CAPs (i.e., X_n×t_ for the FPN states correlating with the neuropsychological score) with n=22*5 rows as the sample size of tumor patients where the network was detected and t =5 columns as the delta of main temporal properties for the FPN-CAP of interest between three-months follow up and baseline (i.e. in-degree, out-degree, occurrences, betweenness centrality and resilience). The other dataset the behavioural attentive/executive variables (i.e., y_n×m_) with n=22 rows as the sample size of tumor patients and m=4 columns as delta of behavioural variable of interest (i.e. TMT and attentional matrices). Both data matrices were normalized column-wise (i.e., z-scored) in order to identify the latent variables. The correlation matrix R=X'Y was then subjected to the following singular value decomposition: R=X'Y=USV' where S_m×m_ is the diagonal matrix of singular values and U_t×m_ and V_m×m_ are the orthonormal matrices of the left and right singular vectors, respectively. A latent variable corresponds to each column in the **U** and **V** matrices. Each element of the diagonal of **S** is the corresponding singular value. The temporal FPN-CAP features' and behavioural features' relative contributions to latent variables are shown by the left and right singular vectors, U and V, respectively.

Positively weighted temporal FPN-CAP features correlate with positively weighted behavioural features, whereas negatively weighted temporal FPN-CAP and behavioural correlate with each other. Brain scores show how much each area of the brain displays the weighted patterns found by latent variables that can be estimated using singular vectors. The computation of brain scores for temporal CAP and behavioural characteristics involves projecting the initial data onto the weights that are determined from PLS, specifically **U** and **V**, obtaining:

- Brain scores for temporal FPN-CAP features = **XU**
- Brain scores for behavioural features= **YV**

The Pearson correlation coefficient between the original data matrices and the relevant brain

scores are then used to calculate loadings for temporal FPN-CAP features and behavioural features. The correlation coefficients between the initial temporal FPN-CAP characteristics vectors and the PLS-derived brain scores for temporal FPN-CAP features, for instance, are known as temporal FPN-CAP features loadings. Using 10000 permutation tests^27^, the statistical significance of latent variables (LC) was evaluated. The original data was randomized using spatial autocorrelation-preserving nulls. Every permutation was subjected to the PLS analysis once more, producing a null distribution of singular values. After that, the original singular values' significance was evaluated in comparison to the permuted null distributions. Using bootstrap resampling, which involves randomly resampling rows of the original data matrices **X** and **Y** 500 times with replacement, the dependability of PLS loadings was assessed. Next, for every resampled data set, the PLS analysis was performed once again to provide a sampling distribution for every temporal FPN-CAP feature and behavioural feature (i.e., 500 bootstrap-resampled loadings). We next utilize the bootstrap-resampled loading distributions to determine the loadings' 95% confidence intervals (e.g., see Figure 2). The decomposition of each LC in the set of behavioral weights and FPN CAP temporal features weights, represent how largely each variable contributes to the multivariate brain-behavior correlations across runs^23^. Moreover, two additional confirmatory PLS analyses were performed respectively between the FPN or the DAN four CAP’s temporal measures and executive score across different groups to understand the overall involvement of networks in predicting cognitive performance. For FPN, results (p=0.005; r=0.81) showed that the relationship with Δ TMT scores is better explained by properties of CAP1_FPN_ and CAP3_FPN_, in addition to CAP4_FPN_ (Figure S1, panels A and B). For DAN, the significant LC (p=0.014; r=0.78) is explained by higher Δ in CAP1_DAN_ in-degree, without specific association to any cognitive score (Figure S1, panels C and D).

Linear models

Linear models (*lm*, R-package, *lme4*, R-package) were used to investigate the prediction of attentional and executive profile from the time-varying connectivity of the FPN. In what follows each model is described by introducing each response and relative predictors. The included random intercept term was kept constant across models to account for individual variability, allowing each subject to have a unique baseline level and thereby capturing the unobserved heterogeneity across subjects. Statistical threshold of significance was set to p<0.05, after correcting for multiple comparisons with FDR across estimates of interests.

1. Model 1: Linear mixed model

The first model was used to test the hypothesis that dynamic longitudinal properties of FPN could predict the longitudinal attentive and executive performance of the patients. Therefore, for each attentional score (attentional matrices, TMT-A, TMT-B and TMT-BA) the following model was applied, using pre-surgical (as T0) and three months follow-up data (as T1). The presence of postsurgical attentional deficit was defined as a categorical variable described in the main text.

*Attentional scores ~ Time × Dynamic network properties × Attentional deficit postsurgical presence + (1|Subject)*

Results of the model are displayed in Table S3.

1. Model 2: Linear model

The second model was used to test the hypothesis that presurgical dynamic properties of FPN could predict the presence of attentional deficit in the immediate post-surgical timepoint. Therefore, by considering the presence of postsurgical attentional deficit (defined as a categorical variable described in the main text) as response, the following model was applied, using pre-surgical data only.

*Attentional deficit postsurgical presence ~ Presurgical dynamic network properties*

Results of the model are displayed in Table S4.

1. Model 3: Linear model

The third model was used to test the hypothesis that presurgical dynamic properties of FPN could predict attentional and executive pre-surgical performance. Therefore, for each attentional score (attentional matrices, TMT-A, TMT-B and TMT-BA) the following model was applied, using pre-surgical data only.

*Presurgical attentional scores ~ Presurgical dynamic network properties*

Results of the model are displayed in Table S5.

1. Model 4: Linear model

The fourth model was used to test the hypothesis that presurgical dynamic properties of FPN could predict immediate post-surgical attentional and executive performance. Therefore, for each attentional score (attentional matrices, TMT-A, TMT-B and TMT-BA) the following model was applied, using pre-surgical data only for the MRI data and immediate post-surgical data for the neuropsychological part.

*Postsurgical attentional scores ~ Presurgical dynamic network properties*

Results of the model are displayed in Table S6.

1. Model 5: Linear model

The fifth model was used to test the hypothesis that presurgical dynamic properties of FPN could predict three months post-surgical attentional and executive performance. Therefore, for each attentional score (attentional matrices, TMT-A, TMT-B and TMT-BA) the following model was applied, using pre-surgical data only for the MRI data and three months post-surgical follow up data for the neuropsychological part.

*Follow-up attentional scores ~ Presurgical dynamic network properties*

Results of the model are displayed in Table S7.

**References**

1. Saviola F, Zigiotto L, Novello L, et al. The role of the default mode network in longitudinal functional brain reorganization of brain gliomas. *Brain Struct Funct*. April 2022. doi:10.1007/s00429-022-02490-1
2. Catricalà, E., della Rosa, P.A., Ginex, V., Mussetti, Z., Plebani, V., Cappa, S.F., 2012. An Italian battery for the assessment of semantic memory disorders. Neurological Sciences 2012 34:6 34, 985–993. <https://doi.org/10.1007/S10072-012-1181-Z>
3. Papagno, C., Casarotti, A., Zarino, B., Crepaldi, D., 2020. A new test of action verb naming: normative data from 290 Italian adults. Neurological Sciences 41, 2811–2817. <https://doi.org/10.1007/S10072-020-04353-1/TABLES/4>
4. Novelli, G., Papagno, C., Capitani, E., Laicona, M., Vallar, G., Cappa, S.F., 1986. Tre test clinic di ricerca e produzione lessicale. Taratura su soggetti normali. Archivio di Psicologia Neurologia e psichiatria 4, 477–506.
5. Orsini, A., Grossi, D., Capitani, E., Laiacona, M., Papagno, C., Vallar, G., 1987. Verbal and spatial immediate memory span: Normative data from 1355 adults and 1112 children. The Italian Journal of Neurological Sciences 8, 537–548.
6. Carlesimo, G.A., Caltagirone, C., Gainotti, G., Fadda, L., Gallassi, R., Lorusso, S., Marfia, G., Marra, C., Nocentini, U., Parnetti, L., 1996. The Mental Deterioration Battery: Normative Data, Diagnostic Reliability and Qualitative Analyses of Cognitive Impairment. European Neurology 36, 378–384.
7. Caffarra, P., Vezzadini, G., Dieci, F., Zonato, F., Venneri, A., 2002a. Rey-Osterrieth complex figure: normative values in an Italian population sample. Neurological Sciences 22, 443–447.
8. Casarotti, A., Papagno, C., Zarino, B., 2014. Modified Taylor Complex Figure: Normative data from 290 adults. Journal of Neuropsychology 8, 186–198. <https://doi.org/10.1111/JNP.12019>
9. Spinnler, H., Tognoni, G., 1987. Taratura e standardizzazione italiana di test neuropsicologici. Italian Journal of Neurological Sciences 8, 8–120.
10. Giovagnoli, A.R., del Pesce, M., Mascheroni, S., Simoncelli, M., Laiacona, M., Capitani, E., 1996. Trail making test: normative values from 287 normal adult controls. The Italian Journal of Neurological Sciences 17, 305–309.
11. Caffarra, P., Vezzadini, G., Dieci, F., Zonato, F., Venneri, A., 2002b. Una versione abbreviata del test di Stroop: dati normativi nella popolazione italiana. Nuova Rivista di Neurologia 12, 111–115.
12. Mancuso, M., Rosadoni, S., Capitani, D., Bickerton, W.L., Humphreys, G.W., de Tanti, A., Zampolini, M., Galardi, G., Caputo, M., de Pellegrin, S., Angelini, A., Bartalini, B., Bartolo, M., Carboncini, M.C., Gemignani, P., Spaccavento, S., Cantagallo, A., Zoccolotti, P., Antonucci, G., 2015. Italian standardization of the Apples Cancellation Test. Neurological Sciences 36, 1233–1240
13. Fortis, P., Maravita, A., Gallucci, M., Ronchi, R., Grassi, E., Senna, I., Olgiati, E., Perucca, L., Banco, E., Posteraro, L., Tesio, L., Vallar, G., 2010. Rehabilitating patients with left spatial neglect by prism exposure during a visuomotor activity. Neuropsychology 24, 681–697.
14. Basso, A., Capitani, E., Laiacona, M., 1987. Raven’s coloured progressive matrices: normative values on 305 adult normal controls. Functional Neurology 2, 189–194.
15. Zigiotto L, Annicchiarico L, Corsini F, et al. Effects of supra-total resection in neurocognitive and oncological outcome of high-grade gliomas comparing asleep and awake surgery. *J Neurooncol*. 2020;148(1):97-108. doi:10.1007/s11060-020-03494-9
16. Dallabona M, Sarubbo S, Merler S, et al. Impact of mass effect, tumor location, age, and surgery on the cognitive outcome of patients with high-grade gliomas: A longitudinal study. *Neuro-Oncology Pract*. 2017;4(4):229-240. doi:10.1093/nop/npw030
17. Capitani E, Laiacona M. Aging and psychometric diagnosis of intellectual impairment: Some considerations on test scores and their use. *Dev Neuropsychol*. 1988;4(4):325-330. doi:10.1080/87565648809540416
18. Papagno, C., Casarotti, A., Comi, A. et al. Measuring clinical outcomes in neuro-oncology. A battery to evaluate low-grade gliomas (LGG). J Neurooncol 108, 269–275 (2012). <https://doi.org/10.1007/s11060-012-0824-5>
19. Zigiotto L, Vavassori L, Annicchiarico L, et al. Segregated circuits for phonemic and semantic fluency: A novel patient-tailored disconnection study. *NeuroImage Clin*. 2022;36:103149. doi:10.1016/j.nicl.2022.103149
20. Zigiotto L, Annicchiarico L, Corsini F, et al. Effects of supra-total resection in neurocognitive and oncological outcome of high-grade gliomas comparing asleep and awake surgery. *J Neurooncol*. 2020;148(1):97-108. doi:10.1007/s11060-020-03494-9
21. Sarubbo S, De Benedictis A, Merler S, et al. Towards a functional atlas of human white matter. *Hum Brain Mapp*. 2015;36(8):3117-3136. doi:10.1002/hbm.22832
22. Sarubbo S, Tate M, De Benedictis A, et al. Mapping critical cortical hubs and white matter pathways by direct electrical stimulation: an original functional atlas of the human brain. *Neuroimage*. 2020;205:116237. doi:10.1016/j.neuroimage.2019.116237
23. Zöller D, Sandini C, Karahanoğlu FI, et al. Large-Scale Brain Network Dynamics Provide a Measure of Psychosis and Anxiety in 22q11.2 Deletion Syndrome. *Biol Psychiatry Cogn Neurosci Neuroimaging*. 2019;4(10):881-892. doi:10.1016/j.bpsc.2019.04.004
24. Monti, S., Tamayo, P., Mesirov, J. et al. Consensus Clustering: A Resampling-Based Method for Class Discovery and Visualization of Gene Expression Microarray Data. Machine Learning 52, 91–118 (2003). https://doi.org/10.1023/A:1023949509487
25. Chen JE, Chang C, Greicius MD, Glover GH. Introducing co-activation pattern metrics to quantify spontaneous brain network dynamics. *Neuroimage*. 2015;111:476-488. doi:10.1016/j.neuroimage.2015.01.057
26. Bolton TAW, Tuleasca C, Wotruba D, et al. TbCAPs: A toolbox for co-activation pattern analysis. *Neuroimage*. 2020;211:116621. doi:10.1016/j.neuroimage.2020.116621
27. Bommarito G, Tarun A, Farouj Y, et al. Altered anterior default mode network dynamics in progressive multiple sclerosis. Multiple Sclerosis Journal. 2022;28(2):206-216. doi:10.1177/13524585211018116

**Supplementary Tables**

Table S1: Age and education adjusted attention and executive cognitive scores of glioma patients at different time points of their intervention (Pre: pre-surgical, Post: immediate post-surgical, FU 3 m: follow-up at 3 months). The last column shows the % of patients with cognitive pathological scores in each test (i.e., relative to the normal population).

| **^Cognitive Tests^** | **^Time^** | **^Mean cognitive score (SD)^** | **^% of patients with deficit^** |
| --- | --- | --- | --- |
| ^Attentional matrices^  ^(selective attention)^ | ^Pre^ | ^76.3 (±13.1)^ | ^4.5 %^ |
|  | ^Post^ | ^73.7 (±17.5)^ | ^13.64 %^ |
|  | ^FU 3 m^ | ^77.9 (±12.9)^ | ^0.0 %^ |
| ^Trail Making Test part A^  ^(divided attention)^ | ^Pre^ | ^30.8 (±19.7)^ | ^4.5 %^ |
|  | ^Post^ | ^44.0 (±28.8)^ | ^13.6 %^ |
|  | ^FU 3 m^ | ^31.1 (±12.0)^ | ^0.0 %^ |
| ^Trail Making Test part B^  ^(alternating attention, mental/cognitive flexibility)^ | ^Pre^ | ^100.4 (±59.7)^ | ^4.5 %^ |
|  | ^Post^ | ^159.0 (±96.6)^ | ^18.2 %^ |
|  | ^FU 3 m^ | ^94.9 (±51.9)^ | ^0.0 %^ |
| ^Trail Making Test part B - A^  ^(alternating attention, mental/cognitive flexibility)^ | ^Pre^ | ^69.5 (±42.6)^ | ^4.5 %^ |
|  | ^Post^ | ^112.9 (±79.8)^ | ^27.3 %^ |
|  | ^FU 3 m^ | ^64.3 (±44.2)^ | ^0.0 %^ |

Table S2: **Bootstrap data corresponding to Figure 2**: PLSC results from attentional cognitive outcome through fronto-parietal dynamism. The table shows bootstrap mean and 5 to 95 percentiles of behavior weights and brain weights.

| **Weights type** | **Item** | **Mean** | **5^th^ percentile** | **95^th^ percentile** |
| --- | --- | --- | --- | --- |
| **Behaviour:**  **Attentive performence** | ∆TMTA (Normal)  ∆TMTA (Deficit) | -0.28  -0.90 | -0.73  -0.99 | 0.31  -0.58 |
|  | ∆TMTB (Normal)  ∆TMTB (Deficit) | -0.98  0.18 | -0.99  -0.52 | -0.95  0.74 |
|  | ∆TMTB-A (Normal)  ∆TMTB-A (Deficit) | -0.38  -0.87 | -0.98  -0.99 | 0.69  0.29 |
|  | ∆Attentional matrices (Normal)  ∆Attentional matrices (Deficit) | -0.86  0.30 | -0.99  -0.88 | 0.13  0.93 |
| **CAP4_FPN_ properties** | ∆Occurrences | 0.85 | 0.53 | 0.98 |
|  | ∆Betweeneess centrality | 0.04 | -0.52 | 0.62 |
|  | ∆Resilience | 0.95 | 0.86 | 0.98 |
|  | ∆In-degree | -0.12 | -0.62 | 0.39 |
|  | ∆Out-degree | 0.19 | -0.28 | 0.72 |

Table S3.1: **Model 1**, Executive and attentive neuropsychological profile showing significant effects revealed by a linear mixed model of longitudinal changes (pre-surgical and three-months follow-up) in brain tumor patients stratified into two groups (with or without attentional deficit in the immediate post-surgical time point). The model included the following main predictors: *time* (a positive effect means neuropsychological score increases regardless the presence of attentional deficit and dynamic properties), *attentional deficit* (a positive effect means neuropsychological score increases regardless the time and dynamic properties), *time × attentional deficit* (a positive effect means that longitudinal neuropsychological score increases faster in patients with attentional deficit relative to normal one). The model also included dynamic functional connectivity scores of the Fronto-Parietal network reported in other tables. Significant (p < 0.05) fixed effects are emphasized in bold and an asterisk.

| ***Response*** | ***Predictors*** *[Estimate* β*, p-value]* | | | ***Random effects*** |
| --- | --- | --- | --- | --- |
|  | *Time* | *Attentional Deficit* | *Time* × *Attentional Deficit* | σ^2^ |
| ***Attentional matrices*** | [-0.3, 0.9] | [-8.1, 0.1] | [2.4, 0.6] | 17.9 |
| ***TMT-A*** | [-0.6, 0.9] | [10.2, 0.2] | [-9.2, 0.2] | 55.9 |
| ***TMT-B*** | [80.8, 0.7] | [71.3, **0.0004]*** | [-32.7, **0.04**] | 272.4 |
| ***TMT-BA*** | [52.5,0.7] | [60.8, **0.001**]* | [-23.3, 0.1] | 159.4 |

Table S3.2: **Model 1**, Executive and attentive neuropsychological profile showing significant effects revealed by a linear mixed model of longitudinal changes (pre-surgical and three-months follow-up) in brain tumor patients stratified into two groups (with or without attentional deficit in the immediate post-surgical time point). The model included the following main predictors of dynamic functional connectivity patterns of the Fronto-Parietal Network: *betweenness centrality; occurrences, in degree, out degree and resilience.* For all of them, a positive effect means neuropsychological score increases regardless of time, other dynamic properties and the presence of attentional deficit. The model also included time and attentional deficit presence reported in other tables. Significant (p < 0.05) fixed effects are emphasized in bold and an asterisk.

| ***Response*** | ***Predictors*** *[Estimate* β*, p-value]* | | | | | ***Random effects*** |
| --- | --- | --- | --- | --- | --- | --- |
|  | *Betweenness centrality* | *Occurrences* | *In Degree* | *Out Degree* | *Resilience* | σ^2^ |
| ***Attentional matrices*** | [0.5, 0.4] | [0.1, 0.4] | [-259.6, 0.1] | [-192.7, 0.2] | [-73.6, 0.6] | 17.9 |
| ***TMT-A*** | [-1.5, 0.2] | [-0.2, 0.1] | [229.9, 0.4] | [219.5, 0.4] | [415.9, 0.1] | 55.9 |
| ***TMT-B*** | [-4.9, **0.035]*** | [-0.2, 0.6] | [605.2, 0.4] | [600.9, 0.3] | [362.4, 0.5] | 272.4 |
| ***TMT-BA*** | [-3.9, **0.032]*** | [0.1, 0.7] | [292.6, 0.6] | [340.5, 0.4] | [-110.6, 0.8] | 159.4 |

Table S3.3: **Model 1**, Executive and attentive neuropsychological profile showing significant effects revealed by a linear mixed model of longitudinal changes (pre-surgical and three-months follow-up) in brain tumor patients stratified into two groups (with or without attentional deficit in the immediate post-surgical time point). The model included the following interaction predictors of longitudinal dynamic functional connectivity patterns of the Fronto-Parietal Network: *betweenness centrality × time, occurrences × time, in degree × time, out degree × time and resilience × time.* For all of them, a positive effect means that longitudinal neuropsychological score increases faster in time regardless of the presence of the attentional deficit. The model also included time and attentional deficit presence predictors reported in other tables. Significant (p < 0.05) fixed effects are emphasized in bold and an asterisk.

| ***Response*** | ***Predictors*** *[Estimate* β*, p-value]* | | | | | ***Random effects*** |
| --- | --- | --- | --- | --- | --- | --- |
|  | *Time* × *Betweenness centrality* | *Time* × *Occurrences* | *Time* × *In Degree* | *Time* × *Out Degree* | *Time* × *Resilience* | σ^2^ |
| ***Attentional matrices*** | [0.6, 0.4] | [-0.1, 0.5] | [380.9, 0.1] | [253.9, 0.2] | [32.4, 0.8] | 17.9 |
| ***TMT-A*** | [0.9, 0.6] | [0.2, 0.5] | [13.7, 0.9] | [17.8, 0.9] | [-324.7, 0.3] | 55.9 |
| ***TMT-B*** | [2.0, 0.6] | [0.5, 0.4] | [-1047.3, 0.2] | [-937.0, 0.2] | [-424.9, 0.5] | 272.4 |
| ***TMT-BA*** | [1.1, 0.7] | [0.2, 0.7] | [-817.0, **0.02**]* | [-782.9, 0.2] | [2568.9, **0.002**]* | 159.4 |

Table S3.4: **Model 1**, Executive and attentive neuropsychological profile showing significant effects revealed by a linear mixed model of longitudinal changes (pre-surgical and three-months follow-up) in brain tumor patients stratified into two groups (with or without attentional deficit in the immediate post-surgical time point). The model included the following interaction predictors of longitudinal dynamic functional connectivity patterns of the Fronto-Parietal Network: *betweenness centrality × attentional deficit, occurrences × attentional deficit, in degree × attentional deficit, out degree × attentional deficit and resilience × attentional deficit.* For all of them, a positive effect means that longitudinal neuropsychological score increases faster in patients with attentional deficit relative to normal ones regardless of time. The model also included time and attentional deficit presence predictors reported in other tables. Significant (p < 0.05) fixed effects are emphasized in bold and an asterisk.

| ***Response*** | ***Predictors*** *[Estimate* β*, p-value]* | | | | | ***Random effects*** |
| --- | --- | --- | --- | --- | --- | --- |
|  | *Attentional Deficit* × *Betweenness centrality* | *Attentional Deficit* × *Occurrences* | *Attentional Deficit* × *In Degree* | *Attentional Deficit* × *Out Degree* | *Attentional Deficit* × *Resilience* | σ^2^ |
| ***Attentional matrices*** | [0.6, 0.6] | [0.4, 0.1] | [-299.6, 0.3] | [253.9, 0.2] | [-365.8, 0.2] | 17.9 |
| ***TMT-A*** | [-5.0, **0.027]*** | [-1.3, **0.001]*** | [1726.2, **0.001]*** | [1819.4, **0.001]*** | [955.3, **0.047]*** | 55.9 |
| ***TMT-B*** | [0.5, 0.9] | [-3.4, **0.001]*** | [2725.6, **0.0017]*** | [2589.9, **0.013]*** | [3420.3, **0.002**]* | 272.4 |
| ***TMT-BA*** | [2568.9, **0.002]*** | [-2.2, **0.001]*** | [1183.2, 0.2] | [914.5, 0.3] | [2568.9, **0.002**]* | 159.4 |

Table S3.5: **Model 1**, Executive and attentive neuropsychological profile showing significant effects revealed by a linear mixed model of longitudinal changes (pre-surgical and three-months follow-up) in brain tumor patients stratified into two groups (with or without attentional deficit in the immediate post-surgical time point). The model included the following interaction predictors of longitudinal dynamic functional connectivity patterns of the Fronto-Parietal Network: *betweenness centrality × attentional deficit × time, occurrences × attentional deficit × time, in degree × attentional deficit × time, out degree × attentional deficit × time and resilience × attentional deficit ×.* For all of them, a positive effect means that longitudinal neuropsychological score increases faster in patients with attentional deficit relative to normal ones during time. The model also included time and attentional deficit presence predictors reported in other tables. Significant (p < 0.05) fixed effects are emphasized in bold and an asterisk.

| ***Response*** | ***Predictors*** *[Estimate* β*, p-value]* | | | | | ***Random effects*** |
| --- | --- | --- | --- | --- | --- | --- |
|  | *Time* × *Attentional Deficit* × *Betweenness centrality* | *Time* × *Attentional Deficit* × *Occurrences* | *Time* × *Attentional Deficit* × *In Degree* | *Time* × *Attentional Deficit* × *Out Degree* | *Time* × *Attentional Deficit* × *Resilience* | σ^2^ |
| ***Attentional matrices*** | [-1.7, 0.4] | [-0.5, 0.2] | [49.3, 0.9] | [134.4, 0.7] | [562.6, 0.2] | 17.9 |
| ***TMT-A*** | [1.5, 0.7] | [2.9, **0.001]*** | [-1993.2, **0.003]*** | [-2206.3, **0.001]*** | [-2848.1, **0.001]*** | 55.9 |
| ***TMT-B*** | [-15.6, **0.047]*** | [3.7, **0.007]*** | [254.2, 0.9] | [1114.8, 0.4] | [-4518.1, **0.005**]* | 272.4 |
| ***TMT-BA*** | [-16.7, **0.006]*** | [0.8, 0.4] | [1913.7, 0.1] | [3064.5, **0.005]*** | [-1718.8, **0.156**]* | 159.4 |

Table S4: **Model 2**, The presence of attentional deficit showing significant effects revealed by a linear model of dynamic temporal properties of the Fronto-parietal network. The model included the following main predictors of dynamic functional connectivity patterns of the Fronto-Parietal Network: *betweenness centrality; occurrences, in degree, out degree and resilience.* For all of them, a positive effect means neuropsychological score increases regardless of other dynamic properties. Significant (p < 0.05) fixed effects are emphasized in bold and an asterisk.

| ***Response*** | ***Predictors*** *[Estimate* β*, p-value]* | | | | |  |
| --- | --- | --- | --- | --- | --- | --- |
|  | *Betweeness centrality* | *Occurrences* | *In Degree* | *Out Degree* | *Resilience* | ***R^2/^/ R^2^ adjusted*** |
| ***Attentional deficit*** | [-0.1, 0.5] | [-0.0, 0.9] | [31.3, **0.0009]*** | [21.7, **0.040]*** | [7.2, 0.5] | 0.2/0.1 |

Table S5: **Model 3**, Pre-surgical neuropsychological scores showing significant effects revealed by a linear model of pre-surgical dynamic temporal properties of the Fronto-parietal network. The model included the following main predictors of dynamic functional connectivity patterns of the Fronto-Parietal Network: *betweenness centrality; occurrences, in degree, out degree and resilience.* For all of them, a positive effect means pre-surgical neuropsychological score increases regardless of other pre-surgical dynamic properties. Significant (p < 0.05) fixed effects are emphasized in bold and an asterisk.

| ***Response*** | ***Predictors*** *[Estimate* β*, p-value]* | | | | |  |
| --- | --- | --- | --- | --- | --- | --- |
|  | *Betweeness centrality* | *Occurrences* | *In Degree* | *Out Degree* | *Resilience* | ***R^2^/ R^2^ adjusted*** |
| ***Attentional matrices*** | [1.6, **0.045]*** | [0.3, **0.035]*** | [-637.6, **0.002]*** | [-517.1, **0.004]*** | [-289.3, 0.09] | 0.2/0.2 |
| ***TMT-A*** | [-3.8, 0.1] | [-1.0, **0.005]*** | [1504.3, **0.003]*]** | [1255.7, **0.005]*** | [1139.9, **0.009]*** | 0.2/0.2 |
| ***TMT-B*** | [-7.3, 0.2] | [-2.5, **0.023]*** | [5322.9, **0.001]*** | [4034.1, **0.003]*** | [2098.2, 0.1] | 0.2/0.2 |
| ***TMT-BA*** | [-3.9, 0.4] | [-1.3, 0.1] | [3716.3, **0.001]*** | [2716.8, **0.005]*** | [820.9, 0.4] | 0.2/0.2 |

Table S6: **Model 4**, Post-surgical neuropsychological scores showing significant effects revealed by a linear model of pre-surgical dynamic temporal properties of the Fronto-parietal network. The model included the following main predictors of dynamic functional connectivity patterns of the Fronto-Parietal Network: *betweenness centrality; occurrences, in degree, out degree and resilience.* For all of them, a positive effect means post-surgical neuropsychological score increases regardless of other pre-surgical dynamic properties. Significant (p < 0.05) fixed effects are emphasized in bold and an asterisk.

| ***Response*** | ***Predictors*** *[Estimate* β*, p-value]* | | | | |  |
| --- | --- | --- | --- | --- | --- | --- |
|  | *Betweeness centrality* | *Occurrences* | *In Degree* | *Out Degree* | *Resilience* | ***R^2^/ R^2^ adjusted*** |
| ***Attentional matrices*** | [0.9, 0.4] | [0.2, 0.3] | [-945.3, **0.001]*** | [-673.9, **0.004]*** | [-12.1, 0.9] | 0.2/0.2 |
| ***TMT-A*** | [-0.4, 0.9] | [0.1, **0.883]*** | [1602.4, **0.038]*** | [1045.4, **0.123]*** | [-637.4, 0.4] | 0.1/0.1 |
| ***TMT-B*** | [-3.9, 0.4] | [-1.3, 0.1] | [5314.1, **0.001]*** | [3716.3, 0.2] | [-2027.5, 0.4] | 0.1/0.7 |
| ***TMT-BA*** | [-1.3, 0.8] | [-0.3, 0.7] | [2848.0, **0.016]*** | [1932.7, 0.1] | [-422.9, 0.7] | 0.2/0.1 |

Table S7: **Model 5**, Three-months follow-up neuropsychological scores showing significant effects revealed by a linear model of pre-surgical dynamic temporal properties of the Fronto-parietal network. The model included the following main predictors of dynamic functional connectivity patterns of the Fronto-Parietal Network: *betweenness centrality; occurrences, in degree, out degree and resilience.* For all of them, a positive effect means follow-up neuropsychological score increases regardless of other pre-surgical dynamic properties. Significant (p < 0.05) fixed effects are emphasized in bold and an asterisk.

| ***Response*** | ***Predictors*** *[Estimate* β*, p-value]* | | | | |  |
| --- | --- | --- | --- | --- | --- | --- |
|  | *Betweenness centrality* | *Occurrences* | *In Degree* | *Out Degree* | *Resilience* | ***R^2^/ R^2^ adjusted*** |
| ***Attentional matrices*** | [1.0, 0.2] | [0.0, 0.9] | [-182.3, 0.4] | [-167.7, 0.4] | [21.8, 0.9] | 0.0/-0.0 |
| ***TMT-A*** | [0.3, 0.8] | [-0.0, 0.9] | [315.3, 0.4] | [192.5, 0.5] | [-96.9, 0.7] | 0.0/-0.0 |
| ***TMT-B*** | [-1.3, 0.8] | [-0.4, 0.7] | [3251.9, **0.020]*** | [2203.1, 0.1] | [-444.4, 0.7] | 0.1/0.1 |
| ***TMT-BA*** | [-1.3, 0.8] | [-0.3, 0.7] | [2848.0, **0.016]*** | [1932.6, 0.1] | [-422.9, 0.7] | 0.1/0.1 |

Table S8: **Anatomical lobe localization of the tumor across participants**

| Tumor lobe location (N) | Tumor hemisphere (R, L) | Tumor grade (HGG, LGG) |
| --- | --- | --- |
| Frontal (7) | (4,3) | (5,2) |
| Fronto-parietal (1) | (1,0) | (1,0) |
| Fronto-temporal (2) | (2,0) | (2,0) |
| Fronto-mesial (1) | (0,1) | (0,1) |
| Insula (1) | (1,0) | (1,0) |
| Occipital (1) | (1,0) | (0,1) |
| Parietal (3) | (1,2) | (2,1) |
| Temporal (5) | (1,4) | (2,3) |
| Temporo-mesial (1) | (0,1) | (0,1) |

**Supplementary Figures**

**Figure S1**: A) and B) Significant Latent Component (LC) identified with grouped behavioral Partial Least Square Analysis with all CAPs from the FPN seed explaining differing effects of covariance in patients with different attentive performance disregarding absolute group differences. C) and D) Significant Latent Component (LC) identified with grouped behavioral Partial Least Square Analysis with all CAPs from the FPN seed explaining differing effects of covariance in patients with different attentive performance disregarding absolute group differences.

**^
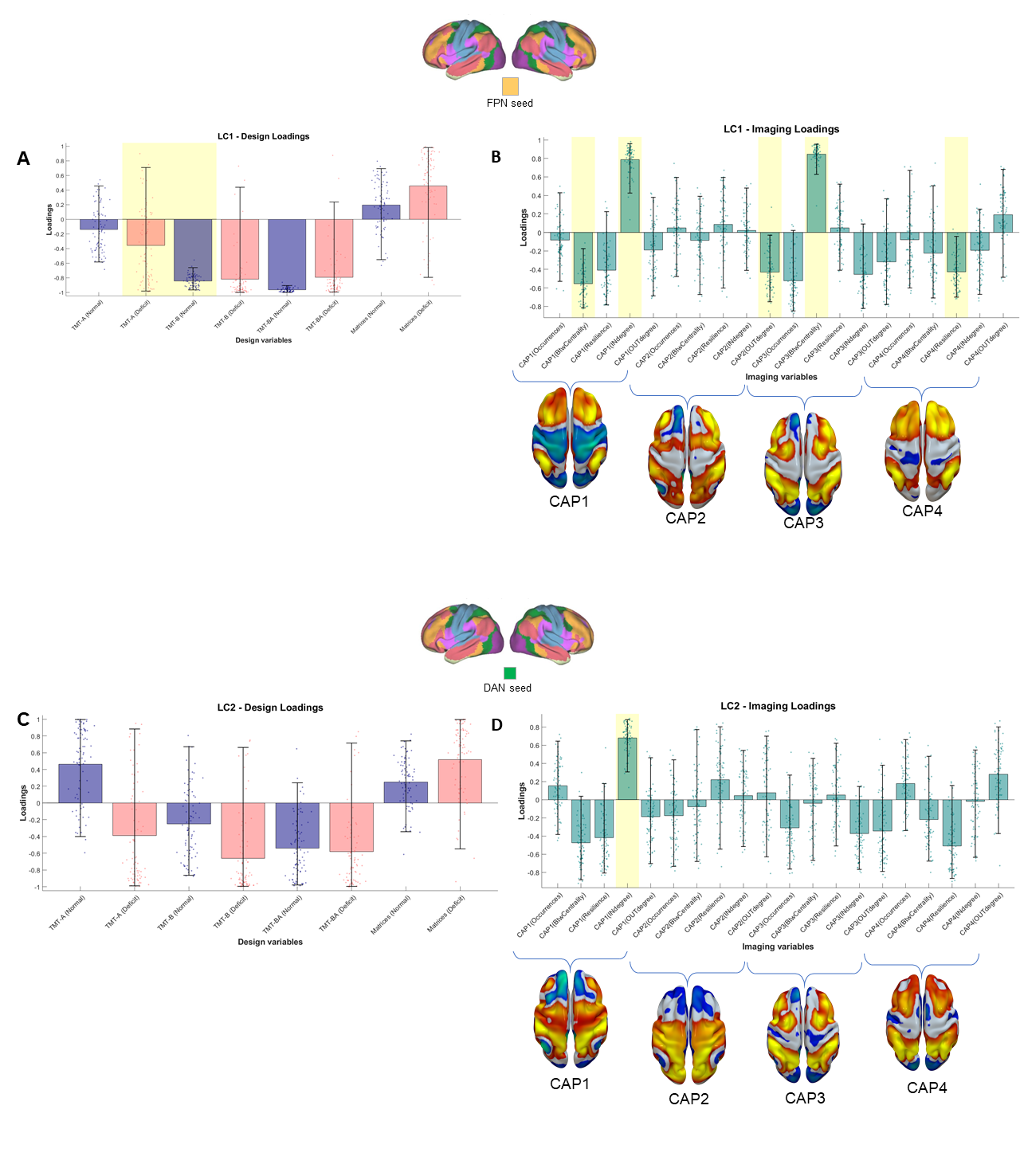
^**

Figure S2: **Prediction of longitudinal attentive/executive scores through longitudinal fronto-parietal temporal properties for Model 1.**

**^
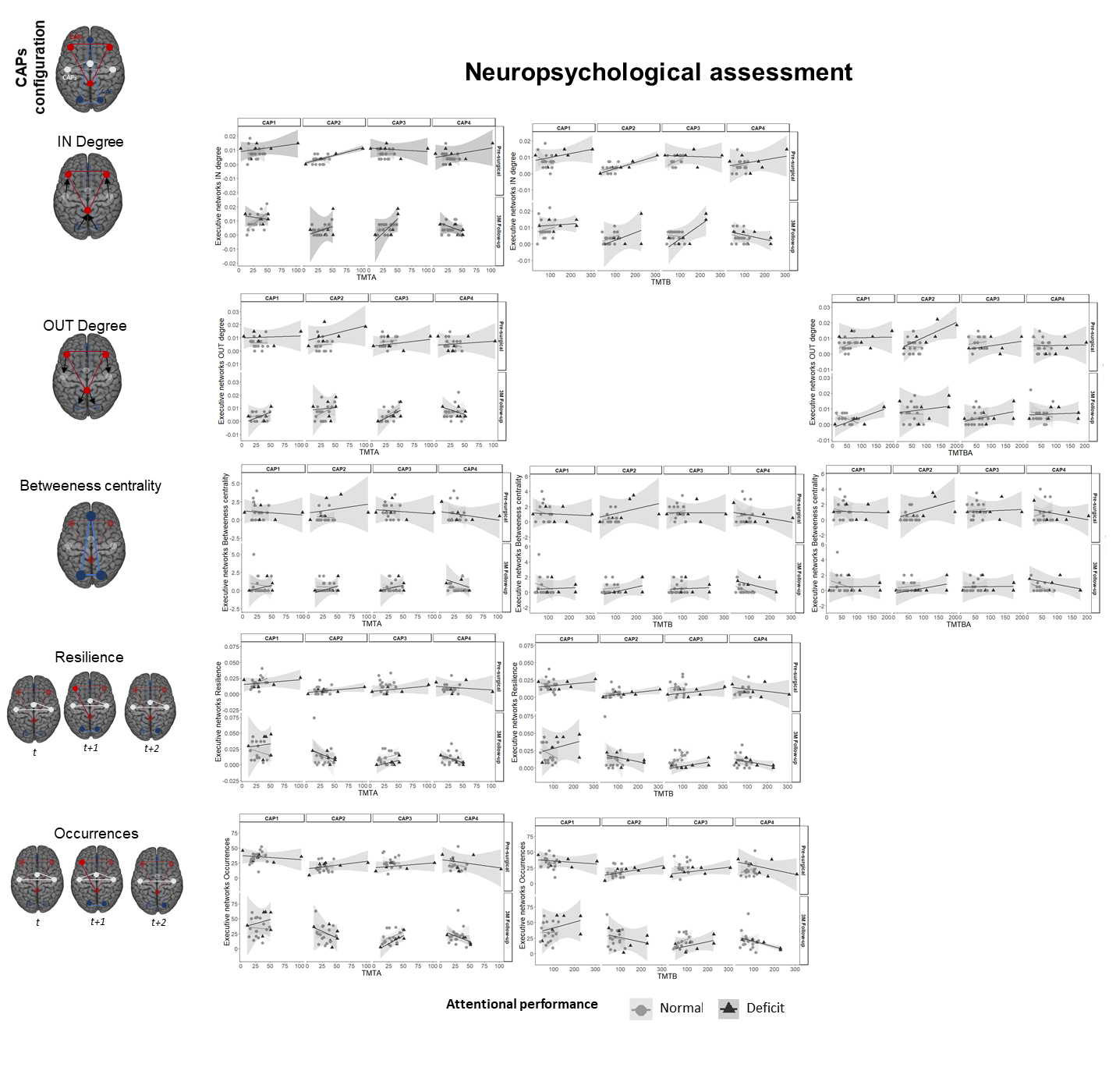
^**


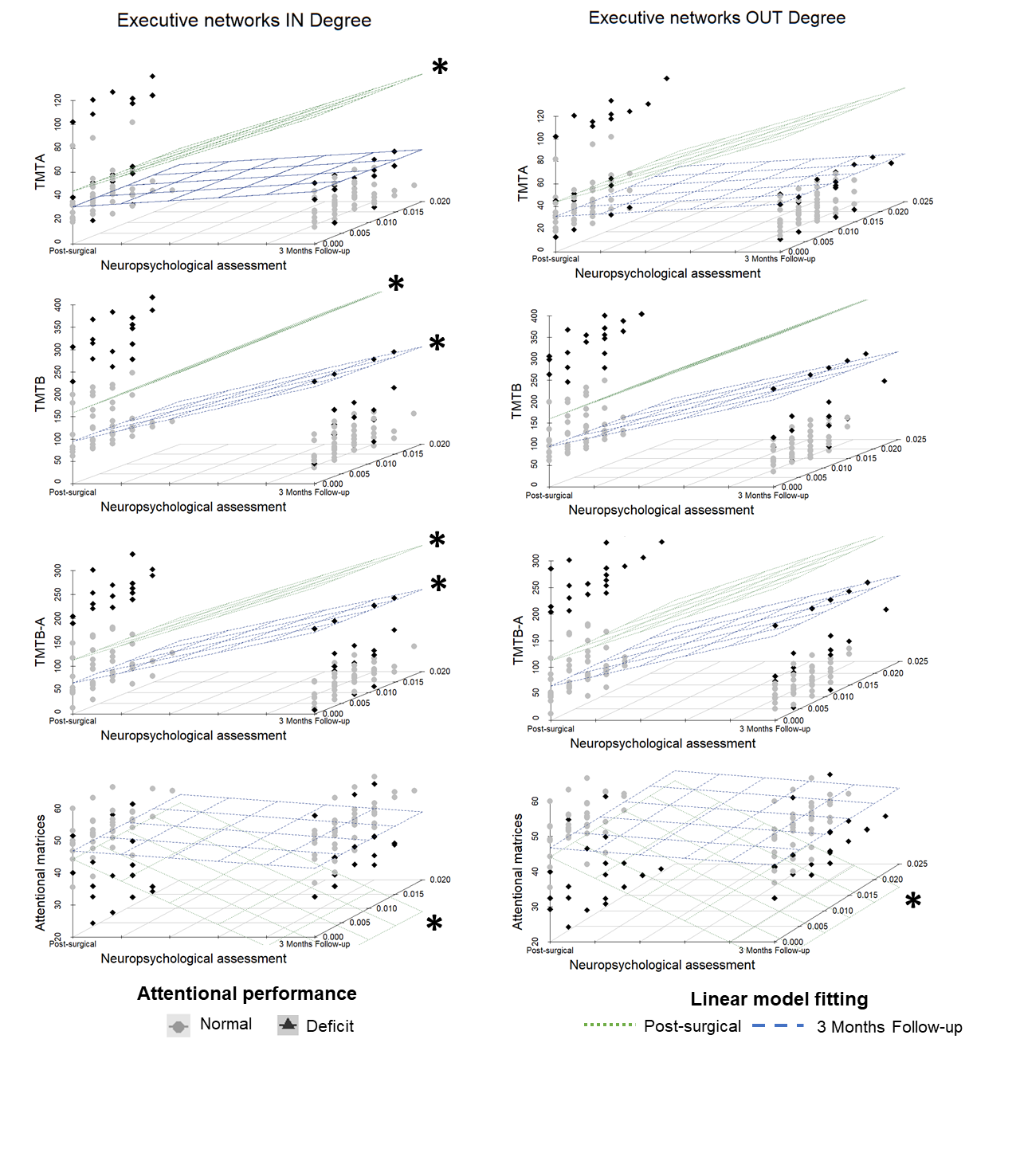
Figure S3**: Prediction of postoperative (immediate and 3 months follow-up) attentional cognitive outcome through pre-surgical fronto-parietal temporal properties.** 3D Scatterplots of linear models investigating respectively if presurgical dynamic properties of FPN could predict immediate post-surgical (model 4) or three-months follow-up (model 5) attentional and executive performance, displayed as a function of pre-surgical IN Degree properties of FPN (probability of transitions from other dynamics states to the FPN, first column) and pre-surgical OUT Degree properties of FPN (probability of transition from the FPN towards other dynamic states, second column) for each neuropsychological test. Significant model effect visualized as 3D plane are marked with *. The *Deficit* subgroup are patients with at least one deficit in the neuropsychological battery at 1-week post-surgery discharge, the *Normal* group has no deficits.

Figure S4**: Probability map of tumors distribution together with Frontoparietal (FPN) and Dorsal Attention (DAN) networks seeds.**


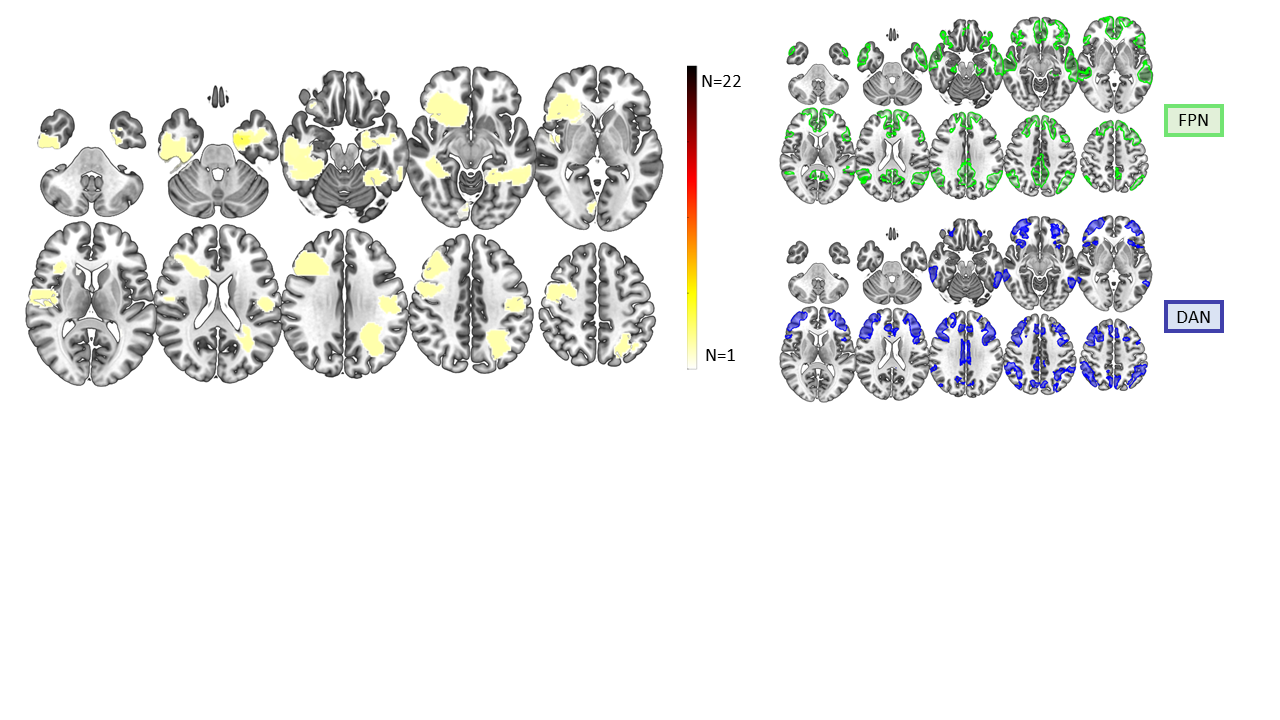

Supplement: Supplementary file 1 — Supplementary Material 1 [file 11060_2025_5079_MOESM1_ESM.docx]
